# A Study on the Applicability of Thermodynamic Sensors in Fermentation Processes in Selected Foods

**DOI:** 10.3390/s22051997

**Published:** 2022-03-03

**Authors:** Martin Adamek, Jiri Matyas, Anna Adamkova, Jiri Mlcek, Martin Buran, Martina Cernekova, Veronika Sevcikova, Magdalena Zvonkova, Petr Slobodian, Robert Olejnik

**Affiliations:** 1 Department of Physics and Materials Engineering, Faculty of Technology, Tomas Bata University in Zlin, Vavreckova, 275, 760 01 Zlin, Czech Republic; m2adamek@utb.cz; 2 Department of Microelectronics, Faculty of Electrical Engineering and Communication, Brno University of Technology, Technicka, 3058/10, 616 00 Brno, Czech Republic; martin.buran@vutbr.cz; 3 Centre of Polymer Systems, University Institute, Tomas Bata University in Zlin, Trida Tomase Bati, 5678, 760 01 Zlin, Czech Republic; slobodian@utb.cz (P.S.); olejnik@utb.cz (R.O.); 4 Department of Food Analysis and Chemistry, Faculty of Technology, Tomas Bata University in Zlin, Vavreckova, 275, 760 01 Zlin, Czech Republic; aadamkova@utb.cz (A.A.); mlcek@utb.cz (J.M.); v2_sevcikova@utb.cz (V.S.); m1_zvonkova@utb.cz (M.Z.); 5 Department of Fat, Surfactant and Cosmetics Technology, Faculty of Technology, Tomas Bata University in Zlin, Vavreckova, 275, 760 01 Zlin, Czech Republic; cernekova@utb.cz; 6 Polymer Centre, Faculty of Technology, Tomas Bata University in Zlin, Vavreckova, 275, 760 01 Zlin, Czech Republic

**Keywords:** fermentation, thermodynamic sensors, yeast

## Abstract

This study focuses on the use of thermodynamic sensors (TDS) in baking, brewing, and yogurt production at home. Using thermodynamic sensors, a change in the temperature flow between the two sensor elements during fermentation was observed for the final mixture (complete recipe for pizza dough production), showing the possibility of distinguishing some phases of the fermentation process. Even during the fermentation process in the preparation of wort and yogurt with non-traditional additives, the sensors were able to indicate significant parts of the process, including the end of the process. The research article also mentions as a new idea the use of trivial regulation at home in food production to determine the course of the fermentation process. The results presented in this article show the possibility of using TDS for more accurate characterization and adjustment of the production process of selected foods in the basic phase, which will be further applicable in the food industry, with the potential to reduce the cost of food production processes that involve a fermentation process.

## 1. Introduction

Fermentation is one of the oldest methods of preserving food, and people have used it for thousands of years. It is a natural process of converting organic substances, in which substances of plant and animal origin are transformed with the help of microorganisms (bacteria, yeast, etc.) into simpler substances. Fermentation is used in the food industry, for example, in the production of alcoholic beverages, yeast, fermented sausages, vinegar, and fermented milk products such as kefir, skyr, or cottage cheese, as well as in cheese maturation, dough leavening, and vegetable fermentation [1]. During some fermentation processes, alcohol or lactic or acetic acid is formed, which are natural preservatives that naturally extend the shelf life of food [1,2].

Fermentation has become a trend in world gastronomy. Fermented vegetables (cucumbers, cabbage) are often prepared in households, but the domestic production of sour milk products (yoghurts, kefir), as well as sourdough bread, is also modern. During the fermentation process, foods are obtained that are easily digestible and better usable by the human body. Fermentation is also used to increase the nutritional value of a food, for example, by producing biologically active substances, vitamin C and B, or by improving protein digestibility. On the contrary, antinutrients decrease [1,3]. With the advent of alternative preservation methods, fermentation is also a way to improve the sensory and technological properties of food [4].

There are many types of fermentation, but the most commonly used are alcoholic and lactic. In addition, there is fermentation using noble molds to make certain cheeses, tempeh, or soy sauce; acetic fermentation, which produces acetic acid from ethanol; or propionic fermentation, which is used to make cheeses with meshes.

Alcoholic fermentation is a biochemical process in which plant carbohydrates are converted into alcohol, specifically ethanol, and carbon dioxide in the presence of yeast enzymes, generating energy and heat. Alcoholic fermentation also occurs during the leavening of dough, when yeast enzymes convert carbohydrates into carbon dioxide and ethanol, which ensure the leavening of the dough both during fermentation and during baking. This occurs in a pastry with yeast or yeast alone. In addition to leavening, alcoholic fermentation serves to improve the taste of the resulting pastry. However, lactic fermentation also occurs when the dough is leavened, with the yeast and lactic acid bacteria supporting each other [5].

Lactic fermentation is the process of converting carbohydrates into lactic acid using lactic acid bacteria. If only lactic acid is produced during lactic fermentation, it is a homofermentative lactic fermentation, which is used in the preservation of cabbage or cucumbers, because it prevents the development of putrefactive bacteria. If lactic fermentation produces other products in addition to lactic acid, this is heterofermentative lactic fermentation. The most common products are acetic acid, ethanol, hydrogen, and carbon dioxide [6,7].

Modern and fast methods to determine the phase of the fermentation process include, for example, the frequently used detection of produced fermentation gases using an electronic nose [8,9], or electrochemical impedance spectroscopy [10,11]. One possible method is the use of thermodynamic sensors (TDS). The widespread use of TDS in the food industry has already been indicated on the basis of several experimental procedures, e.g., in flour fermentation monitoring, where fermentation was monitored by both methods (TDS and electronic nose) in samples enriched with grape marc or flour from edible insects [12]. On the same principle, yeast viability can also be monitored more quickly [13]; this would traditionally be measured using relatively slow culture methods [14,15,16] or more sophisticated analytical methods, such as fluorescent-labelled flow cytometry. However, this method requires very expensive equipment [17]. Of course, with the need to speed up monitoring, new methods such as TDS or the use of a portable microscope based on the principle of optical fibers are being developed [18].

However, the use of thermodynamic sensors is not limited to yeast fermentation in the bakery. They can also be used to monitor the fermentation of lactic acid bacteria in dairy products. Dairy products are an integral part of rational nutrition. In particular, due to the presence of probiotic bacteria, they may have a prophylactic effect on the development of some diseases [19]. Already, the first experiments with TDS showed their promising use (high sensitivity to changes in temperature flow, the ability to monitor some phases of the fermentation process without direct contact with fermented food, low cost) [20], which was also confirmed by repeated use of TDS for dairy fermentation monitoring [13]. Another modern method for monitoring the fermentation process uses high-throughput monitoring of the pH, which, while maintaining accuracy (compared to conventional electrodes for pH measurement), gives the opportunity to shorten the analysis time [21]. Another option is to monitor dielectric properties [22]. Various types and modifications of spectroscopic methods are also frequently used to study the fermentation of dairy products [23,24,25,26].

Another possible application of TDS is monitoring in brewing and viticulture. TDS may also find potential use as a control mechanism against the growth of unwanted microflora [27].

The above-mentioned publications on the use of TDS in the food industry dealt with incomplete studies, focused on the one specific example of applicability. More comprehensive studies expanding knowledge of the applicability of TDS in monitoring the fermentation of other common and less traditional raw materials are still lacking. There is also a lack of deeper knowledge of the TDS response when monitoring more complete units such as mixtures composed of multiple ingredients. The aim of this study was therefore to supplement some of this missing information and to show other possibilities of a low-cost way to monitor the fermentation process of three different types of food—pizza dough with insect flour, brewing in malt production, and dairy in yogurt with added non-traditional commodities (insect flour from dark flour and flour from dried goji berry).

## 2. Materials and Methods

### 2.1. Materials

The experiment was divided into 3 parts according to the area of use—monitoring the fermentation process in the production of dough (a recipe for pizza dough was used), beer, and yogurt.

During the fermentation of the dough, the system was initially tested by monitoring the fermentation of baker’s yeast. The ingredients used for the experiment are listed in Table 1.

The ingredients listed in Table 2 were used to ferment the basic pizza dough.

Different proportions of edible insects were used to fortify the dough. The basic material was a powder (flour) made from the larvae of (*Tenebrio molitor*). Larvae in the developmental phase just before pupation were used. The larvae were purchased at Radek Frýželka, Brno, Czech Republic. The larvae were removed from the farm, displaced for 48 h, and killed using boiling water (100 °C). They were further dried at 105 °C, homogenized, and stored in a refrigerator at 4–7 °C until further measurement. The content of insect flour and the weights of individual raw materials for sample production are given in Table 3.

In the second part, the monitoring of top fermentation of malt for beer production in domestic brewing was monitored. For this reason, the following were used:

- Upper dried yeast of Ale beer, Safale S-04, Fermentis, Lesaffre Group, Marcq-en-Barœul, France, 11.5 g;
- Maltose LIGHT (Malt extract—dried), Mr. Sládek s.r.o., Pivovar-Šenov, Obecní 6, 739 34 Šenov, the Czech Republic, 1 kg;
- Drinking water from the common water supply system of the city of Brno, the Czech Republic, 7 L.

In the case of yoghurt fermentation, the ingredients are listed in Table 4. For the sample without additives, only the Lactoflora and organic milk were used. During the fortification, flour from edible insects or dried goji berry fruits (*Lycium chinense*) was added to the above composition of the base mixture.

### 2.2. Methods

Testing of the experimental measuring system at the beginning of this work was done during the monitoring of the fermentation of baker’s yeast. A container of water and beet sugar was placed in the measuring system. After a short period of temperature stabilization, baker’s yeast was poured into the vessel, and the mixture was stirred. Subsequently, the box was closed for complete isolation from the environment throughout the measurement. The sample was monitored every 5 s for approximately 1.5 h. Sample analysis was performed with simple temperature control at 35 ± 2 °C. Each sample was analyzed two times (Experiments E1 and E2) in two thermodynamic systems S1 and S2 (four characteristics in total).

During the fermentation of the dough, the sample (mixture of flour with additives, 40 g; drinking water, 20 mL; sugar, 0.6 g; salt, 0.6 g; olive oil, 2.4 mL; and dried yeast, 0.04 g) was placed in a plastic measuring container, mixed thoroughly, and then placed in a special isolation box for analysis. The output voltage values of the thermodynamic system were recorded for at least 1.5 h at intervals of one second.

For wort fermentation, 1 kg of maltose was mixed into 6 L of water. Dried top yeast was chosen to shorten the test time in this experiment. The yeast was mixed in 1 L of water and poured into the prepared maltose solution. The resulting wort was poured into a 10 L fermentation vessel and placed in a thermally insulated box with a controlled internal temperature of 17 ± 1 °C. Monitoring was performed for more than 85 h, and samples were taken at regular intervals of 2 min.

In the last part, the fermentation of yogurts was monitored. The procedure was similar to that in the first bakery yeast fermentation test. The measuring container with milk was preheated in a water bath to a temperature of about 35 °C and placed in the measuring system. After a short period of temperature stabilization, the dried yogurt culture was poured into a vessel, and the mixture was stirred. Subsequently, the box was closed for complete isolation from the environment throughout the measurement. The sample was monitored every 15 s for a minimum of 7.5 h. The analysis of the sample was performed with simple temperature control at 35 ± 2° C. Each sample was analyzed twice in two thermodynamic systems (four characteristics in total). An exception was the preparation of yogurt with the addition of goji berry, when the second measurement did not take place under reproducible conditions.

### 2.3. Experimental Measuring Equipment

**Figure 1:**
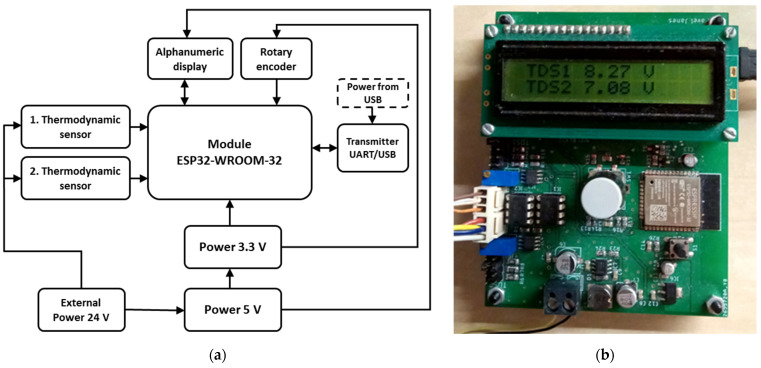
Experimental equipment: (**a**) Block diagram; (**b**) Prototype board of the measuring unit.

The basis of the instrumentation was an experimental device that allows for measurements on the principle of thermodynamic balance, referred to as a thermodynamic sensor (TDS). The principle of the device is described in detail in [10,20]. The prototype described in [28] was used as the basic experimental device. A block diagram and a photograph of the prototype board are shown in Figure 1.

**Figure 2:**
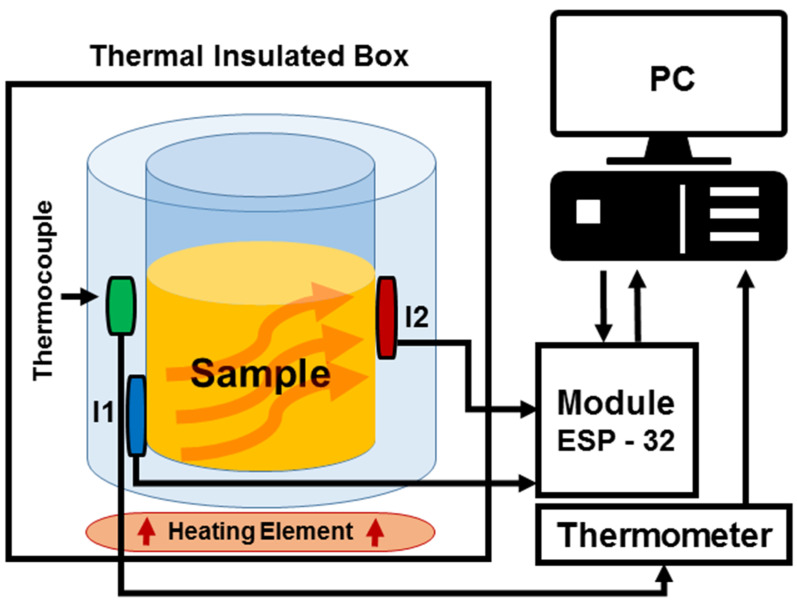
The scheme of construction of the thermodynamic system with the experimental device.

The prototype of the measuring unit allows for setting and measuring two systems of thermodynamic sensors at the same time. Although the electronic system processes both measured channels S1 and S2 in the same way (the change can only be in the feedback settings), the actual mechanical design and conditions of the temperature sensors (Figure 2) on the measured object may differ slightly for systems connected to individual channels of the measuring unit, despite efforts to produce completely identical measuring systems. This general disadvantage affected the sensitivity of the individual systems used, and in the experiments described in this article, the thermodynamic system connected to channel S2 of the measuring unit was more sensitive than that connected to channel S1. However, a comparison showed that the nature of the measured signals was similar.

The measuring system outputs to an LCD alphanumeric display and allows data to be sent directly via Wi-Fi or USB. However, the system can also be autonomous, where data are stored in the memory of the ESP32-WROOM-32 module, which controls the prototype. A rotary encoder was used to control the device. The program allows for sampling at set intervals (1, 5, 15, 30, and 60 s) and is supplemented by the function of averaging the output voltage of sensors in a given period; however, this was not used in this article. The supply voltage of the experimental equipment was 20 V; therefore, the possible output voltage of the equipment was in the range of 1–19 V. The prototype was used for the part monitoring the fermentation of dough and yogurt. Only the basic analogue part of the measuring system was used to monitor the fermentation process in beer production, the output of which was read using an Almemo 2930-5 data logger (Ahlborn Mess-und Regelungstechnik GmbH, Holzkirchen, Germany) and recorded using ALMEMO^®^ Control 6.2.0 (Ahlborn Mess-und Regelungstechnik GmbH, Holzkirchen, Germany). A thermocouple was also connected to the data logger, which sensed the temperature of the plastic fermentation vessel, which was placed in a thermostatic insulation measuring box.

## 3. Results and Discussion

### 3.1. System Testing Using Baker’s Yeast

**Figure 3:**
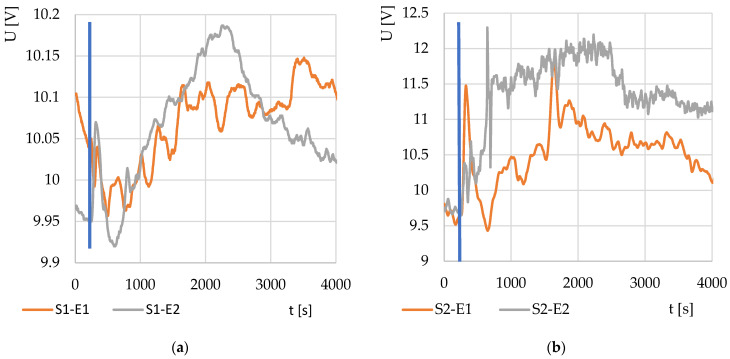
Testing of the device on a model solution of yeast (1 g) in water (150 mL) with beet sugar (2.5 g) for (**a**) thermodynamic system S1 and (**b**) thermodynamic system S2. The experiment was performed twice (E1 and E2).

Thermodynamic systems S1 and S2 were tested on a model solution of baker’s yeast in water with sugar. The results of the two tests are shown in Figure 3. The different output voltages are due to the different design characteristics of the systems.

### 3.2. Monitoring Pizza Dough Leavening

**Figure 4:**
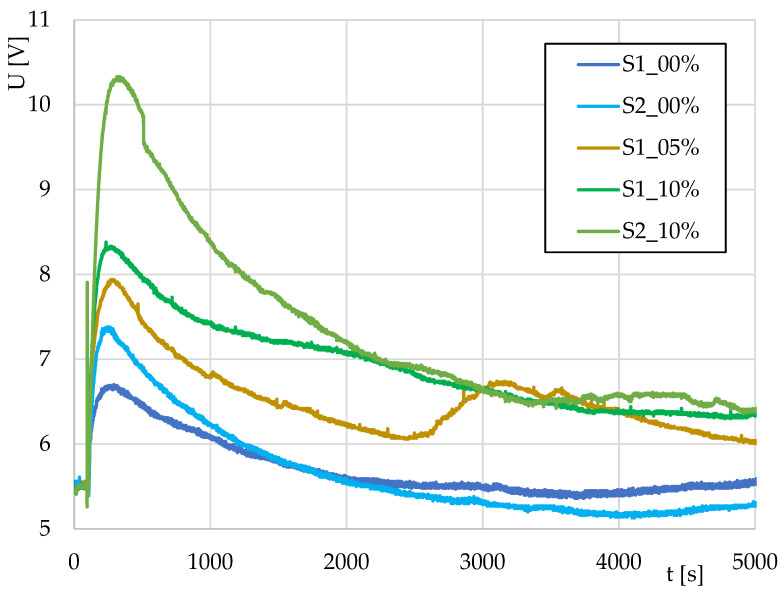
Monitoring of pizza dough leavening without (S1_00%; S2_00%) and with the addition of 5% (S1_05%) or 10% (S1_10%; S2_10%) flour from *Tenebrio molitor*.

The first part of the work was focused on monitoring the leavening of dough without (0%) and with (5% and 10%) the addition of dark flour. The results are shown in Figure 4. The curve plotting the fermentation had a similar course in all three samples. In the first stage, there was a short sharp growth, and in the next part, the curve gradually decreased. The fastest and steepest start of fermentation was detected in the case of a mixture with the addition of 10% insect meal. The values for the sample with the addition of 5% insect meal remained between those for the samples without insect meal and with the addition of 10% insect meal. About halfway through the monitoring, there was an increase and a subsequent decrease, which was caused by a measurement error. In the final phase, the 5% curve stayed above the curve without the additive and that with 10% addition.

### 3.3. Monitoring Beer Fermentation

**Figure 5:**
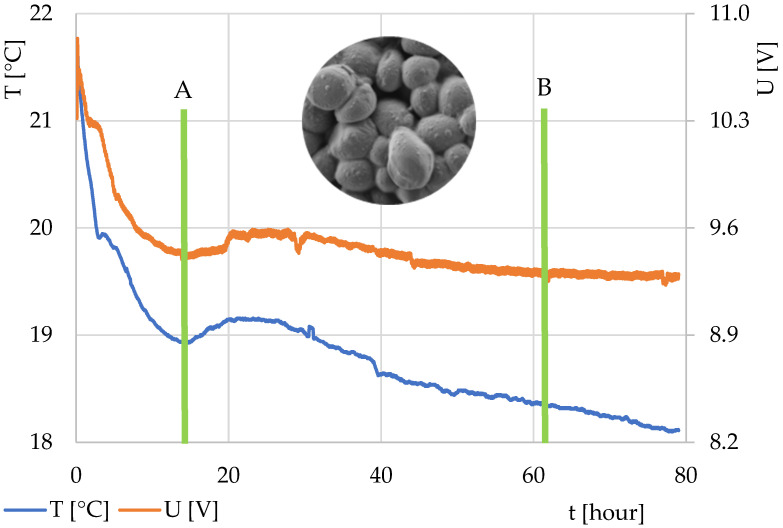
Monitoring process of top fermentation of wort in Experiment No. 1—dried yeast tempered to ambient temperature. Curve description: orange, TDS output voltage; blue, temperature of the plastic fermentation vessel. Lines a and b indicate the beginning and end of the fermentation process. The electron microscope image shows the yeast used in the form of the basic dried material.

**Figure 6:**
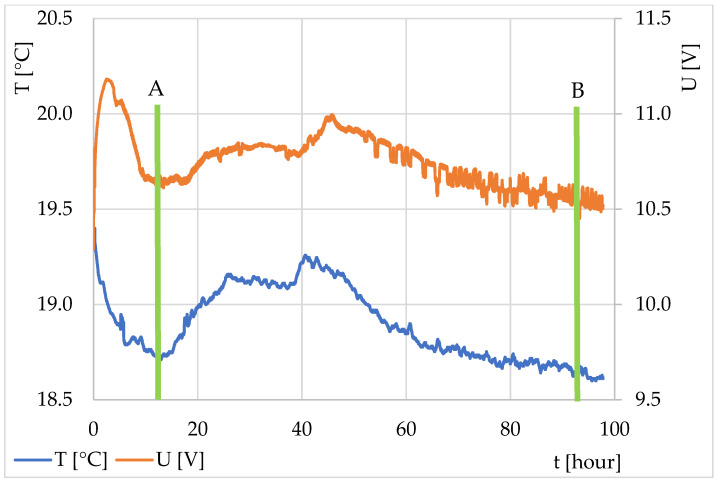
Monitoring of top fermentation of wort in Experiment No. 2—dried yeast directly from the refrigerator. Curve description: orange, TDS output voltage; blue, temperature of the plastic fermentation vessel. Lines a and b indicate the beginning and end of the fermentation process.

In the second part of the work, attention was focused on beer fermentation. The resulting characteristics from the two experiments are shown in Figure 5 and Figure 6. The experiments differed in yeast preparation (Experiment No. 1—yeast tempered to ambient temperature; Experiment No. 2—yeast directly from the refrigerator). The graph in both the experiments began with a decrease, which shows wort cooling and temperature stabilization (equalization of the temperature flow from and to the fermentation mixture, including equalization of the wort temperature and the fermentation batch). The moment at the end of the decrease and the beginning of the rise in the thermodynamic sensor output voltage (in orange in the graph) documents the change in the internal thermal activity and marks the beginning of the fermentation process and an increase in the temperature in the mixture. Alternatively, it might have also meant that the surroundings started to cool down. However, because the entire system was closed and thermally insulated from the environment, this case was not considered. A conventional thermocouple was connected to the outside of the fermentation vessel, the temperature of which is marked in blue in the graph.

The expected end of fermentation occurred when the voltage value from the thermodynamic sensor stabilized and heat flux was absent. This meant that the temperature of the measured solution did not change and had equalized with the ambient temperature. However, this did not mean that the fermentation ended exactly at this time, because the fermentation mixture with the fermentation vessel had a certain heat capacity. This heat capacity of the measuring system caused the accumulation of a certain amount of heat in the system, which caused a reduction in heat flow change in the initial phase of fermentation (part of the heat was used to heat the measuring system) and thermal reverberation of the system at the end of fermentation (when yeast activity had ceased). However, specifying the exact end of fermentation over time would require a different measurement method (usually more expensive with regard to time, materials, or cost) [24].

For such verification measurements, it would be appropriate to use in situ methods, which would not affect the heat fluxes and thermodynamic stability. An example would be a probe or detector that performs analysis in a closed system. Methods for evaluating the relative density of a work on the basis of a change in the buoyancy force at the solution surface are most often used to evaluate the degree of fermentation of the products formed during beer fermentation. Another possible way is an evaluation based on the change in the refraction of electromagnetic waves, i.e., based on refraction [29].

Although initial studies on thermodynamic sensors have been published in the food industry, it is not possible to compare the measured results with the literature directly in terms of collecting and evaluating specific analyte values. TDS sensors can be applied without direct contact with the food being measured, so differences in implementation and differences in operator and financial demands can be discussed. In the case described, however, the main benefit is the described experimental procedure consisting of real-time monitoring of wort fermentation, where the system collects data and stores them every 120 s for almost 100 h. A modern and fast approach applicable in the food industry is the use of biosensors, which, due to the bound component (enzyme, receptor, substrate, etc.), have a high sensitivity for the detection of a given metabolite formed during fermentation [30]. An electronic nose or tongue can also be used in fermentation monitoring, but their widespread acceptance as part of a routine screening method in the food industry is still limited by the insufficient robustness of this method [8,31].

The use of TDS yielded results in previous experiments, where it was verified that the fermentation activity of yeast increases with the addition of sugar, and can also monitor the course of fermentation in the production of yogurt [20]. This was the impetus for the design of further experiments using TDS in the food industry. In another work, the fermentation of wheat flour enriched with grape marc and edible insect flour was evaluated using TDS. Using TDS, by enriching with a small amount of these non-traditional raw materials, we were able to maintain the necessary intensity of fermentation and, at the same time, enrich the product with minerals [10].

Already published results in the measurement of fermentation processes arising during the production of beer deal mainly with contact methods, which are very reliable; however, to a greater extent, they cannot be used at home as they are more economically and professionally demanding. Prior research [32] has dealt with the improvement of observability and the development of a method for active process control of the beer fermentation process. Utilizing a contact method that focuses on the determination of diacetyl, a key biochemical compound during the fermentation process, using a “Cognitive Estimator” model, the Expert System of Fuzzy Logic evaluates the current state of fermentation based on online measured information.

The main difference between the method presented in the present work (TDS) and the method described in the literature [32] is the economic balance and the relatively complex implementation in small and domestic breweries. In contrast to the method of measurement based on data collection from thermodynamic sensors, the fuzzy logic system is very accurate. However, since it works with a large number of conditions and, therefore, with a large number of monitored values from sensors, it can be reasonably assumed that it can be implemented in domestic cooking conditions on more expensive beers.

Prior research [33] has focused on automatic monitoring of the process of continuous fermentation of beer using an automatic membrane input mass spectrometric system. This fermentation monitoring system is accurate but not suitable for home brewing conditions; it requires qualified operation and maintenance of the entire measuring system, as it involves contact measurement of samples with calibration. Another study [34] monitored the fermentation of beer on the principle of hybrid electronic language. In the process of beer production, the analysis of physicochemical parameters is applied, which allows one to determine the phase of the fermentation process and to control its possible failures. It uses a sensor field composed of potentiometric and voltammetric value sensors as the main tool of the study for controlling the brewing process. The aim of the study was to apply an electronic language system to distinguish samples obtained during alcoholic fermentation. The field of sensors applied in the study [34] is a relatively complex system of 10 miniaturized ion-selective electrodes. Setting up the systems in the mentioned references [32,33,34] to measure the fermentation process for all the described methods is not easy, and the systems must be calibrated and operated by a person familiar with the process of brewing. 

### 3.4. Monitoring Yogurt Fermentation with the Addition of Non-Traditional Ingredients

**Figure 7:**
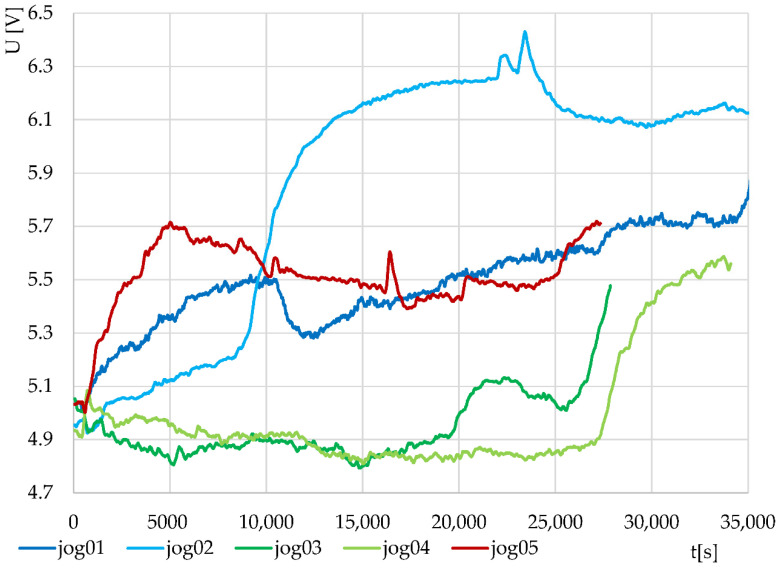
Monitoring of yoghurt fermentation without additives (jog01, jog02), with the addition of edible insect flour (jog03, jog04), and with the addition of goji berry (jog05) for thermodynamic system S1.

**Figure 8:**
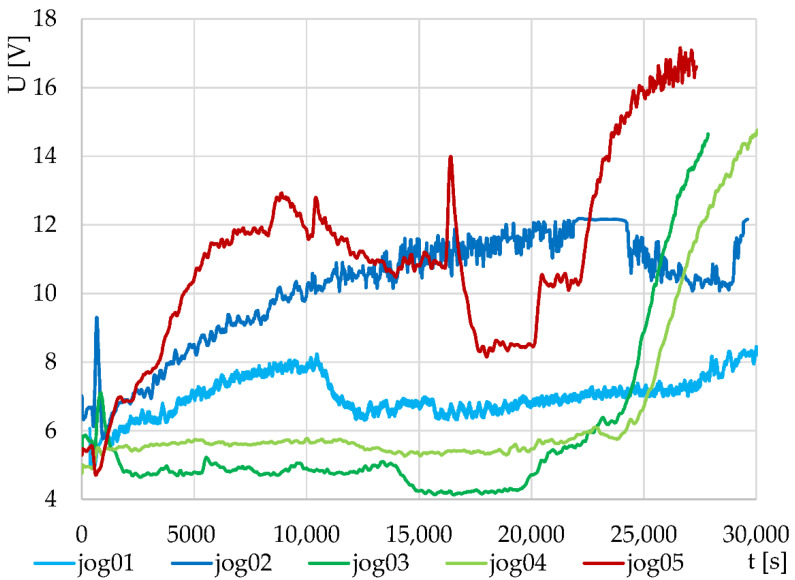
Monitoring of yogurt fermentation without additives (jog01, jog02), with the addition of edible insect flour (jog03, jog04), and with the addition of goji berry (jog05) for thermodynamic system S2.

In the last part, the course of yogurt fermentation was monitored without any additives, with the addition of edible insect flour (5%), and with the addition of goji berry (5%). The resulting graphs for both thermodynamic sensors S1 and S2 are shown in Figure 7 and Figure 8. It was assumed that, similar to its effect in the pastry, the insect meal would support the fermentation rate. However, this phenomenon did not occur in the monitored period of time, and fermentation was additionally suppressed. This phenomenon occurred in all four monitored samples. On the other hand, after the addition of goji berry to the fermented mixture, at the beginning of the fermentation, the fermentation rate increased, as indicated by increased output voltage in both samples. Similar to what occurred in the dough with added insect flour, it is believed that as-yet-undetermined bioactive substances contained in goji berry supported the viability of lactic acid bacteria. Unlike the other measurements, the production of yogurt with the addition of goji berry is not shown in the graph twice, because the second measurement did not have stable and reproducible measuring conditions due to disturbance of the system by the external temperature. However, since two completely separate measuring channels were used and the nature of the signals was similar, it was possible to assume the reliability of these conclusions in the initial phase.

**Figure 9:**
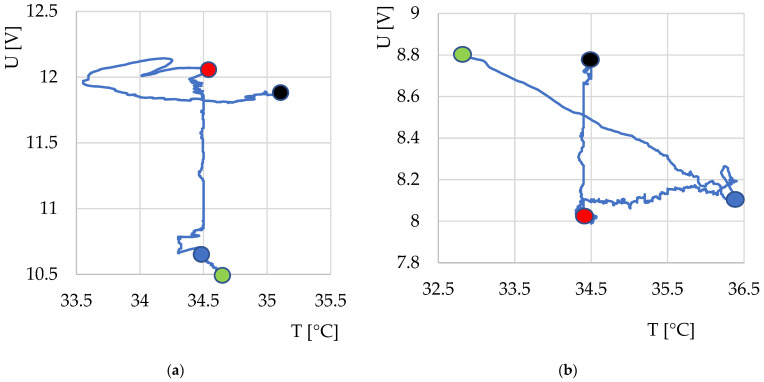
Courses of output voltage and temperature dependences during fermentation monitoring using thermodynamic system S1 for: (**a**) yoghurt without additives; (**b**) yogurt with the addition of insect flour. Description of points: green, start of measurement; blue, insertion of yogurt culture into milk or milk with additives; red, 6 h after the start of measurement (expected time of end of fermentation); black, end of measurement.

Despite efforts to reduce all unwanted thermal effects on sensitive temperature sensors, not all effects could be completely suppressed, and some might have been caused by unwanted interference or a slight shift in the output voltage. However, in an effort to eliminate interfering heat sources, it was found that the direction of the sensor output signal varied depending on the fermentation status and system temperature. Examples of two identified dependencies are shown in Figure 9.

**Figure 10:**
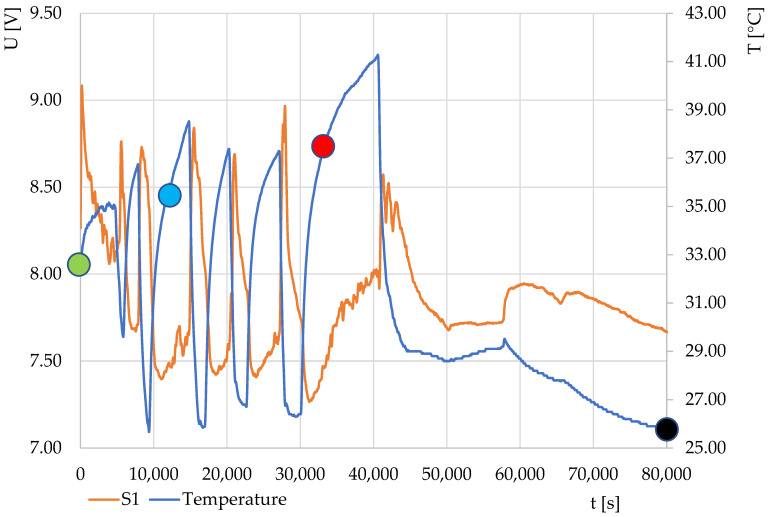
Monitoring the course of yogurt fermentation without additives for thermodynamic system S1 when sweeping the heating temperature. Description of points: green, start of measurement; blue, insertion of yoghurt culture into milk; red, 6 h after the start of measurement (expected time of end of fermentation); black, end of measurement.

**Figure 11:**
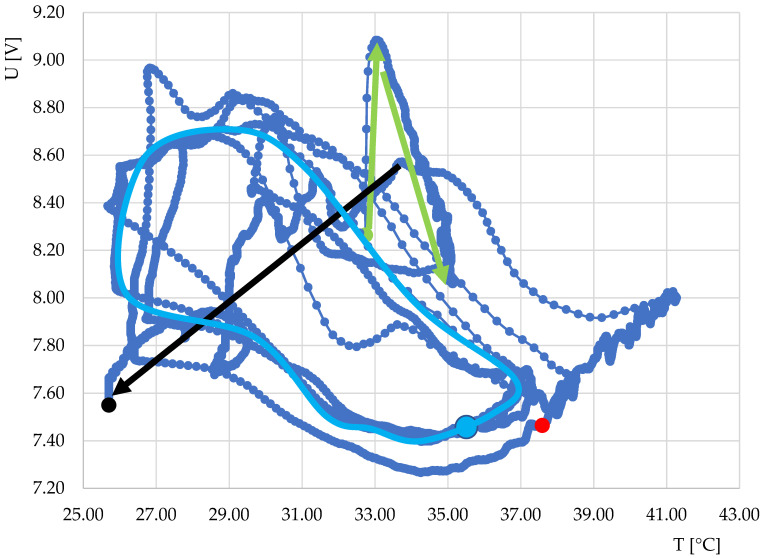
Dependence of output voltage on temperature during individual phases of fermentation in yoghurt production for thermodynamic system S1. Description of the meaning of colors in arrows and points: green point, start of measurement; green arrow, temperature stabilization; blue point, insertion of yogurt culture into milk; blue loop, course of dependence during fermentation; red point, 6 h after start of measurement (estimated time end of fermentation); black arrow, end of fermentation; black point, end of measurement.

Due to the interesting nature of the above curves, the fermentation process in the production of yogurt was monitored, during which the temperature was changed by switching the heating element on and off in the experimental equipment. The resulting course is shown in Figure 10. From the initial data, the characteristics of the output response of sensor S1 during the course of monitoring depending on the change in the heating temperature and the course over time were obtained. The dependence of the output voltage on the temperature is shown in Figure 11. The course of the characteristics shows that after temperature stabilization (marked in green), the characteristics occurred in a loop with a negative direction (indicated by a light blue color). However, after the fermentation, the characteristics changed and the direction changed to positive.

We note that the experiment has not yet been repeated, and without further auxiliary measurements (for example, microbiological determinations), the results cannot be further specified. However, we point out the interesting possibility of using the inaccuracy and hysteresis of thermostats in temperature control, for example, in domestic yogurt makers, to determine the end of the fermentation process.

## 4. Conclusions

This study showed the potential for the use of thermodynamic sensors in baking, brewing, and yogurt production at home. The example measurements showed the possibility of distinguishing some phases of the fermentation process. Using thermodynamic sensors, a change in the temperature flow between the two temperature elements during the leavening of the dough was observed. During the fermentation process in the preparation of wort and yogurt, the sensors were able to warn of the fermentation’s approaching end. This work confirms some results of previously published articles; however, the experiments herein were carried out with raw materials that had not previously been worked with (wort) or on more complete units (a complete recipe for pizza dough production). The work proves that even in these cases it is possible to find and recognize the basic parts of the fermentation process in the resulting time courses. Furthermore, we present as new information the idea of using imperfections in temperature control in simple domestic food equipment to determine the course of the fermentation process.

Monitoring these food preparation steps can bring qualitative and economic benefits, which are in great demand, especially because of the final product, which provides a good ratio of quality and price. Due to the fact that it is a fast, undemanding, and cheap method, we propose its use for further experiments in the food industry, especially to speed up and facilitate analyses, but also to validate the results obtained in the present work. This study was not a study that would be applicable to large food businesses, but its simplicity can help a wider range of people preparing their own products at home to better understand fermentation processes.

## Figures and Tables

**Table 1:** Content of ingredients for tests monitoring the fermentation of baker’s yeast.

Amount	Ingredients	Producer
1 g	Dried yeast	Thymos, spol. s r.o., Veľká Lomnica, Slovakia
2.5 g	Beet sugar	Tereos TTD, Dobrovice, Czech Republic
150 mL	Drinking water	Water supply system of the city of Brno, Czech Republic

**Table 2:** Content of ingredients used to monitor the fermentation of a basic pizza dough.

Amount	Ingredients	Producer
40 g	Smooth wheat flour with a high gluten content “Babiččina volba hladká mouka na kynuté těsto”	GoodMills Česko s.r.o., Prague, Czech Republic
0.6 g	Beet sugar	Tereos TTD, Dobrovice, Czech Republic
0.6 g	Solsanka^®^ Sea salt with iodine and fluorine	Solsan, a.s., Prague, Czech Republic
2.4 mL	Olive oil Franz Josef Kaiser Extra virgin olive oil	GASTON, s.r.o., Zlín, Czech Republic
0.04 g	Gluten-free fresh baker’s yeast brand FALA	Lesaffre Magyarország Ltd., Budapest, Hungary
20 mL	Drinking water	Water supply system of the city of Brno, Czech Republic

**Table 3:** Content of insect flour and weights of individual raw materials for sample production—monitoring dough leavening with the addition of edible insect flour (*Tenebrio molitor*).

Insect Content	0%	5%	10%
Ingredients			
Flour	40 g	38 g	36 g
Flour from edible insects	0 g	2 g	4 g
Water	20 g	20 g	20 g
Sugar	0.6 g	0.6 g	0.6 g
Salt	0.6 g	0.6 g	0.6 g
Olive oil	2.4 mL	2.4 mL	2.4 mL
Yeast	0.04 g	0.04 g	0.04 g

**Table 4:** Content of ingredients for tests monitoring the fermentation of baker’s yeast.

Amount	Ingredients	Producer
150 mL	Organic milk	Olma, a.s., Zábřeh, Czech Republic
1 g	Lactoflora, dried yoghurt for the preparation of sour milk products	Milcom a.s., Prague, the Czech Republic
2.5 g	flour from edible insect or dried fruits	Preparation described in the article

## Data Availability

New research data were presented in this contribution.

## References

[1] Katz S.E. (2012). The Art of Fermentation: An In-Depth Exploration of Essential Concepts and Processes from Around the World.

[2] Cocolin L., Ercolini D. (2008). Molecular Techniques in the Microbial Ecology of Fermented Foods.

[3] Redzepi R., Zilber D. (2018). The Norma Guide to Fermentation. Artisan Division of Workman.

[4] Caplice E., Fitzgerald G. (1991). Food Fermentation: Role of Microorganisms in Food Production and Preservation. Int. J. Food Microbiol..

[5] Struyf N., Van Der Maelen E., Hemdane S., Verspreet J., Verstrepen K.J., Courtin M.C. (2017). Bread Dough and Baker’s Yeast: An Uplifting Synergy. Compr. Rev. Food Sci. Food Saf..

[6] Lahtinen S. (2012). Lactic Acid Bacteria: Microbiological and Functional Aspects.

[7] Hui Y.H., Meunier-Goddik L., Josephsen J., Nip W.-K., Stanfield P.S., Todra F. (2004). Handbook of Food and Beverage Fermentation Technology.

[8] Jiang H., Zhang H., Chen Q., Mei C., Liu G. (2015). Recent advances in electronic nose techniques for monitoring of fermentation process. World J. Microbiol. Biotechnol..

[9] Peris M., Escuder-Gilabert L. (2013). On-line monitoring of food fermentation processes using electronic noses and electronic tongues: A review. Anal. Chim. Acta.

[10] Slouka C., Wurm D.J., Brunauer G., Welzl-Wachter A., Spadiut O., Fleig J., Herwig C. (2016). A Novel Application for Low Frequency Electrochemical Impedance Spectroscopy as an Online Process Monitoring Tool for Viable Cell Concentrations. Sensors.

[11] Brunauer G.C., Meindl A., Rotter B., Gruber A., Slouka C., Schnabel T., Petutschnigg A. (2021). Electrochemical Impedance Spectroscopy for Microbiological Pro-Cesses: On the Way to a Monitoring Tool for the Determination of Biomass. Biomed. J. Sci. Tech. Res..

[12] Adámek M., Adámková A., Mlček J., Vojáčková K., Faměra O., Búran M., Hlobilová V., Bučková M., Baroň M., Sochor J. (2020). Sensor Systems for Detecting Dough Properties Fortified with Grape Pomace and Mealworm Powders. Sensors.

[13] Adámek M., Adámková A., Řezníček M., Kouřimská L. (2016). The estimated possibilities of process monitoring in milk production by the simple thermodynamic sensors. Potravinárstvo.

[14] Jones R.P. (1987). Measures of cell death and deactivation and their meaning: Part I. Process Biochem..

[15] Jones R.P. (1987). Measures of cell death and deactivation and their meaning: Part II. Process Biochem..

[16] Lloyd D., Hayes A.J. (1995). Vigour, vitality and viability of microorganisms. FEMS Microbiol. Lett..

[17] Attfield P.V., Kletsas S., Veal D.A., Van Rooijen R., Bell P.J.L. (2000). Use of flow cytometry to monitor cell damage and predict fermentation activity of dried yeasts. J. Appl. Microbiol..

[18] Wang H., You E., Panneerselvam R. (2021). Advances of surface-enhanced Raman and IR spectroscopies: From nano/microstructures to macro-optical design. Light Sci. Appl..

[19] García-Burgos M., Moreno-Fernández J., Alférez M.J.M., Díaz-Castro J., López-Aliaga I. (2020). New perspectives in fermented dairy products and their health relevance. J. Funct. Foods.

[20] Adámek M., Řezníček M., Adámková A. (2010). The simple thermodynamic sensors for process monitoring in milk production. Electroscope.

[21] Gutsal V., Sieuwerts S., Bibiloni R. (2018). High-throughput pH monitoring method for application in dairy fermentations. J. Dairy Res..

[22] Guo C., Xin L., Dong Y., Zhang X., Wang X., Fu H., Wang Y. (2018). Dielectric Properties of Yogurt for Online Monitoring of Fermentation Process. Food Bioprocess Technol..

[23] Muncan J., Tei K., Tsenkova R. (2021). Real-Time Monitoring of Yogurt Fermentation Process by Aquaphotomics Near-Infrared Spectroscopy. Sensors.

[24] Grassi S., Amigo J.M., Lyndgaard C.B., Foschino R., Casiraghi E. (2014). Beer fermentation: Monitoring of process parameters by FT-NIR and multivariate data analysis. Food Chem..

[25] Mains T.P., Payne F.A., Sama M.P. (2017). Monitoring Yogurt Culture Fermentation and Predicting Fermentation Endpoint with Fluorescence Spectroscopy. Trans. ASABE.

[26] Akiyama M., Ishigaki K., Sakaue S. (2019). Characterizing rare and low-frequency height-associated variants in the Japanese population. Nat. Commun..

[27] Adámková A., Tančinová D., Adámek M. (2013). The Estimated Possibilities of Thermodynamic Sensors in Food Industry.

[28] Jáneš P. (2020). Monitorovací Zařízení pro Kvasné Procesy Využívající Termodynamické Sensory (In Czech, Monitoring Equipment for Fermentation Processes Using the Thermodynamic Sensors). Ph.D. Thesis.

[29] Anton Paar GmbH Beer Fermentation Monitoring. Datasheet D32IA009EN-C. https://www.mtbrandao.com/files/products/D32IA009EN_C_AppRep_Beer_FermentationMonitoring.pdf.

[30] Chandra S., Chapman J., Power A., Roberts J., Cozzolino D. (2015). The application of state-of-the-art analytic tools (biosensors and spectroscopy) in beverage and food fermentation process monitoring. Fermentation.

[31] Mendez M.L., Preedy V. (2016). Electronic Noses and Tongues in Food Science.

[32] Kurz T., Fellner M., Becker A.T., Delgado A. (2001). Observation and Control of the Beer Fermentation Using Cognitive Methods. J. Inst. Brew..

[33] Tarkiainen V., Kotiaho T., Mattila I., Virkajärvi I., Aristidou A., Ketola R.A. (2005). On-line monitoring of continuous beer fermentation process using automatic membrane inlet mass spectrometric system. Talanta.

[34] Kutyła-Olesiuk A., Zaborowski M., Prokaryn P., Ciosek P. (2012). Monitoring of beer fermentation based on hybrid electronic tongue. Bioelectrochemistry.

